# Communication Impairments in Parkinson's Disease

**DOI:** 10.4061/2011/234657

**Published:** 2011-12-18

**Authors:** Bruce Murdoch, Tara Whitehill, Miet de Letter, Harrison Jones

**Affiliations:** ^1^School of Health and Rehabilitation Sciences, The University of Queensland, Brisbane, QLD 4072, Australia; ^2^Division of Speech and Hearing Sciences, The University of Hong Kong, Hong Kong; ^3^Centre of Speech and Hearing Disorders, Ghent University Hospital, 9000 Ghent, Belgium; ^4^Division of Speech Pathology and Audiology, Duke University Medical Center, Durham, NC 27710, USA

A number of different approaches have been utilized in recent years for the treatment of Parkinson's disease with varying effects on associated communication disorders. In particular, the drug treatments based on levodopa therapy and, more recently, deep brain stimulation of the subthalamic nucleus (STN-DBS) have yielded variable effects on the speech disorders exhibited by the majority of persons with Parkinson's disease. With respect to STN-DBS, although reports indicate significant improvement in the symptomatology in Parkinson's disease associated with limb function, the majority of reports suggest that the procedure has either minimal or, in most cases, negative effects on speech abilities of the affected individuals. By far the most effective speech therapy technique applied to dysarthria in Parkinson's disease reported to date is the Lee Silverman Voice Treatment (LSVT) program. In addition to speech impairment, in recent years there has also been increasing awareness that Parkinson's disease is also associated with the occurrence of language disorders, especially in its later stages. This special issue contains 13 papers of which 10 are related to issues associated with speech impairment and 3 further papers explore aspects of language impairment in Parkinson's disease.

The first paper entitled “*Perceived changes in communication as an effect of STN surgery in Parkinson's disease: a qualitative interview study*” by E. Ahlberg et al. explored the perspective of four individuals with Parkinson's disease on the way their speech and communication changed as a result of STN-DBS. Overall the study confirmed earlier findings of negative effects on speech production; however, irrespective of this, the patients in general were positive as to the outcome of their surgery. The paper by F. Karlsson et al. “*Deep brain stimulation of caudal zone incerta and subthalamic nucleus in patients with Parkinson's disease: effects on diadochokinetic rate*,” investigated the outcome of STN-DBS and stimulation of the caudal zone incerta (cZi-DBS) on articulation rate and quality of plosive consonants in two articulatory diadochokinetic tasks. Whereas patients receiving STN-DBS increased in articulation rate with no effect on production quality, patients receiving cZi-DBS decreased in articulation rate and further showed reduction in production quality suggesting that cZi-DBS is more detrimental for extended articulatory movements than STN-DBS.

The third paper, “*Deep brain stimulation of caudal zone incerta and subthalamic nucleus in patients with Parkinson's disease: effects on voice intensity*” by S. Lundgren et al. compares the effects of cZi-DBS and STN-DBS on voice intensity of patients with Parkinson's disease. Overall the results indicated that the two different surgical procedures may have differential effects on voice intensity. The fourth paper by C. Dromey and S. Bjarnason, “*A preliminary report on disordered speech with deep brain stimulation in individuals with Parkinson's disease*,” attempted to determine which laryngeal and articulatory acoustic features changed in patients that reported negative speech outcomes subsequent to STN-DBS. Acoustic measures of articulation and phonation as well as verbal fluency scores showed mixed results with STN-DBS.

The fifth paper in this special issue, “*Effects of LSVT on lexical tone in speakers with Parkinson's disease*” by T. L. Whitehill et al. represents one of the very few studies to have investigated the effect of LSVT on lexical tone. The study involved 12 Cantonese speakers with idiopathic Parkinson's disease who underwent standard LSVT treatment. Although the results showed significant improvement in loudness and intonation after treatment, no significant changes were reported in lexical tone. The sixth paper included in the special issue, “*Acoustic analysis of PD speech*” by K. Chenausky et al. investigated speech changes as a result of STN-DBS settings chosen to maximize motor performance. Importantly, clinically motivated settings evaluated by the researchers showed a greater capacity to impair, rather than improve, speech. Lingual kinematics during consonant production in both dysarthric and nondysarthric speakers with Parkinson's disease were investigated in the seventh paper, “*Lingual kinematics in dysarthric and nondysarthric speakers with Parkinson's disease*” by M. N. Wong et al. The findings indicated that dysarthric and nondysarthric speakers with Parkinson's disease had deviant articulatory movement during consonant production that results in longer duration of consonant production. Importantly, these findings are contrary to contemporary theories that suggest articulatory imprecision in dysarthric speakers with Parkinson's disease is an outcome of reduced range of articulatory movement.

The eighth paper in the current issue, “*Assessment of prosodic communicative efficiency in Parkinson's disease as judged by professional listeners*” by H. Martens et al. investigated the impact of Parkinson's disease on communicative efficiency conveyed through prosody. Although no significant differences were found for emotional prosody, patients with a moderate or severe dysarthria performed significantly worse on imitation of Focus than on reading of Focus. The ninth paper included in the special issue, “*Perception of speech by individuals with Parkinson's disease: a review*” by L. C. Kwan and T. L. Whitehill, explored the possibility of the presence of a deficit in the perception of speech in individuals with Parkinson's disease. Based on a review of the literature, it was concluded that Parkinson's disease specifically impairs patients' perception of verbal emotions. The aim of the tenth paper, “*A cognitive-perceptual approach to conceptualizing speech intelligibility deficits and remediation practice in hypokinetic dysarthria*” by K. L. Landsford et al. was to introduce a novel framework for medical practitioners in order to conceptualize and justify potential targets for speech remediation in patients with Parkinson's disease. The most commonly targeted deficits, including speaking rate and vocal loudness, were supported by this approach.

The eleventh paper, “*High-level language production in Parkinson's disease: a review*” by L. J. P. Altmann and M. S. Troche, reviewed the literature on language production in Parkinson's disease and provided detailed discussion of the language production processes that are impaired in this condition. It was concluded that Parkinson's disease affects all stages of language production, including conceptualization, functional and positional processing. Two semantic priming tasks designed to isolate automatic and controlled semantic activation were utilized in the twelfth paper, “*The influence of dopamine on automatic and controlled semantic activation in Parkinson's disease*” by W. L. Arnott et al. to investigate the impact of dopamine depletion on semantic processing in Parkinson's disease. Overall, the results demonstrated that dopamine depletion can cause disturbances in both automatic and controlled semantic activation. The final paper in this special issue, “*Sentence comprehension and its association with executive functions in patients with Parkinson's disease*” by K. S. F. Colman et al. aimed to provide insight into the interaction between linguistic representation and processing and executive functioning in individuals with Parkinson's disease. Overall, it was concluded that there does not appear to be a language faculty independent of executive functioning.

*Bruce Murdoch* *Bruce Murdoch* *Tara Whitehill* *Tara Whitehill* *Miet de Letter* *Miet de Letter* *Harrison Jones* *Harrison Jones*

